# Supplementary material for: Human papillomavirus genotype distribution among women with and without cervical cancer: Implication for vaccination and screening in Ghana
Source: PLoS One. 2023 Jan 19;18(1):e0280437. doi: 10.1371/journal.pone.0280437 (PMC9851533; doi:10.1371/journal.pone.0280437)
Supplement: Attachment — Submitted filename: Response to reviewer.docx [file pone.0280437.s002.docx]

22^nd^ November, 2022

Dear Dr Aimagambetova,

We thank you for taking the time to carefully read our manuscript and for the valuable comments you have provided, which helped us in improving the revised paper that we are re-submitting. We have made all requested changes, ensured it complies with all the journal requirements.

Yours truly,

Dr Yvonne Nartey

Here is our point-by-point response to the reviewer’s comments:

**(Reviewer comments in bold)**

**This is an well written article on the topic of HPV in Ghana. The analysis presented in the paper are really interesting. I would leave a minor comment on enriching the discussion. In the last paragraph, you might include two or three sentences on Ghana's existing HPV screening program and how your study findings can improve the existing approach.**

Response: We have, accordingly, added the following sentence to the last paragraph of the discussion session.

*“Currently, Ghana do not have a national HPV screening program for cervical cancer. However, there are some public and private health facilities that offer such services. The current study provides useful information on the type of HPV likely to lead to cervical cancer and may help in the triage of women for follow-ups. “ - Page 20, paragraph 1, line 2.*

**Nartey and colleagues present an interesting study on the prevalence of different HPV genotypes among women in Ghana. Studies like this are particularly important in understanding if newer vaccines are needed to treat different strains and the effectiveness of HPV vaccination in other areas. Additional comments are noted below.**

**In line 109, authors state that HPV vaccine effectiveness studies have mainly been conducted among Caucasians. While there is a lack of diversity in research participants in biomedical research, this statement ignores the positive gains made in preventing cervical cancer in multiple countries that has had benefits for people across multiple races. Additionally, this statement seems out of place considering the authors do not mention race and ethnicity throughout the article. The authors should consider either removing this statement are include additional information about how research is needed among different races/ ethnicities and results from this study differ from previous studies conducted among majority Caucasian populations.**

Response: We have, accordingly, revised the sentence to reflect the reviewer comment. The sentence has been altered to the following:

*“The available HPV vaccines have activity against up to nine HPV types (6, 11, 16,18, 31 33 45 52 58)”. – Page5, paragraph 2, line 7.*

**It's unclear if the authors are trying to promote the usage of currently available HPV vaccines in Ghana or if they are requesting a new one. It would be helpful if the authors were clearer about this. Especially in lines 470-471 in the conclusion.**

Response: We have, accordingly, revised the sentence to reflect the reviewer comment. The sentence has been altered to the following:

*“HPV DNA testing for cervical screening and multivalent vaccine targeted at the most prevalent HPV types will be a valuable contribution to Ghana’s cervical cancer control programme than the currently available licensed vaccine.” – page 20, paragraph 2, line 8.*
